# The impact of headache in Europe: principal results of the Eurolight project

**DOI:** 10.1186/1129-2377-15-31

**Published:** 2014-05-21

**Authors:** Timothy J Steiner, Lars Jacob Stovner, Zaza Katsarava, Jose Miguel Lainez, Christian Lampl, Michel Lantéri-Minet, Daiva Rastenyte, Elena Ruiz de la Torre, Cristina Tassorelli, Jessica Barré, Colette Andrée

**Affiliations:** 1 Department of Neuroscience, Norwegian University of Science and Technology, Edvard Griegs Gate, NO-7491 Trondheim, Norway; 2 Division of Brain Sciences, Imperial College London, London, UK; 3 Norwegian National Headache Centre, St Olavs University Hospital, Trondheim, Norway; 4 Department of Neurology, University of Essen, Essen, Germany; 5 Department of Neurology, Hospital Clinico Universitario, University of Valencia, Valencia, Spain; 6 Headache Medical Center Seilerstaette Linz, Department of Neurogeriatric Medicine and Remobilisation, Hospital of the Sisters of Charity, Linz Seilerstaette 4, 4010 Linz, Austria; 7 Departement d’Evaluation et Traitement de la Douleur, Centre Hospitalo-Universitaire de Nice, Nice, France; 8 Lithuanian University of Health Sciences, Kaunas, Lithuania; 9 Asociacion Española de Pacientes con Cefalea (AEPAC), Valencia, Spain; 10 Headache Science Centre, C Mondino National Neurological Institute and Department of Brain and Behaviour, University of Pavia, Pavia, Italy; 11 Center of Public Health Research, CRP-Santé, Strassen, Luxembourg; 12 Department of Pharmaceutical Sciences, University of Basel, Basel, Switzerland

**Keywords:** Headache, Migraine, Impact, Prevalence, Europe, Eurolight project, Global Campaign against Headache

## Abstract

**Background:**

European data, at least from Western Europe, are relatively good on migraine prevalence but less sound for tension-type headache (TTH) and medication-overuse headache (MOH). Evidence on impact of headache disorders is very limited. Eurolight was a data-gathering exercise primarily to inform health policy in the European Union (EU). This manuscript reports personal impact.

**Methods:**

The study was cross-sectional with modified cluster sampling. Surveys were conducted by structured questionnaire, including diagnostic questions based on ICHD-II and various measures of impact, and are reported from Austria, France, Germany, Italy, Lithuania, Luxembourg, Netherlands, Spain and United Kingdom. Different methods of sampling were used in each. The full methodology is described elsewhere.

**Results:**

Questionnaires were analysed from 8,271 participants (58% female, mean age 43.4 y). Participation-rates, where calculable, varied from 10.6% to 58.8%. Moderate interest-bias was detected. Unadjusted lifetime prevalence of any headache was 91.3%. Gender-adjusted 1-year prevalences were: any headache 78.6%; migraine 35.3%; TTH 38.2%, headache on ≥15 d/mo 7.2%; probable MOH 3.1%. Personal impact was high, and included ictal symptom burden, interictal burden, cumulative burden and impact on others (partners and children). There was a general gradient of probable MOH > migraine > TTH, and most measures indicated higher impact among females. Lost useful time was substantial: 17.7% of males and 28.0% of females with migraine lost >10% of days; 44.7% of males and 53.7% of females with probable MOH lost >20%.

**Conclusions:**

The common headache disorders have very high personal impact in the EU, with important implications for health policy.

## Background

The *World Health Report 2001*, published by the World Health Organization (WHO), ranked migraine 19th among the causes of disability worldwide, responsible for 1.4% of all years of life lost to disability (YLDs) [1]. Although this finding has been cited repeatedly, it considerably underreported the disability that migraine imposes on people throughout the world. The reason was lack of good evidence. In the Global Burden of Disease Survey 2000 (GBD2000), on which the *World Health Report 2001* was based, estimates for migraine were derived from very scarce data for China, India and most other countries in South East Asia, most of Africa, all of the Eastern Mediterranean and all of eastern Europe – countries in which more than half the world’s population live. Moreover, GBD2000 gave no account of headache disorders other than migraine: tension-type headache (TTH) and medication-overuse headache (MOH) did not feature, despite contributing significantly to the global disability burden of disease [2]. For these disorders, at that time, dependable evidence was lacking everywhere.

Filling this evidence gap has been a priority of the Global Campaign against Headache [3-11]. In consequence, the Global Burden of Disease Survey 2010 (GBD2010) [12] was much better informed. In this survey, TTH and migraine, with estimated global prevalences of 20.1% and 14.7% respectively, ranked as second and third most common diseases in the world (behind dental caries) in both males and females. Migraine was recognized as the seventh highest among specific causes of disability globally, responsible for 2.9% of all YLDs [12-15].

There are many uncertainties surrounding surveys such as GBD and the rankings they derive as products of prevalence and disability weights. GBD2010 was a much better account of the global burden of headache than GBD2000, but still incomplete [13-15]. It failed to acknowledge the interictal impairments associated with migraine. It altogether ignored MOH. This disorder, which might be regarded as a complication of either migraine or TTH (and should therefore be included in the reckoning of both), undoubtedly causes substantial disability. Nevertheless, GBD2010 confirmed the widespread opinion both among members of the lay headache organizations of Europe and among headache specialists that headache disorders receive far less attention than they deserve as major causes of public ill-health [16], a view very clearly supported by WHO [7].

As far as Europe is concerned, good data exist on migraine prevalence derived from a number of western countries [17,18]. Data are less good for TTH and MOH. With regard to *impact* of headache disorders, evidence is quite limited for migraine [16] and non-existent for TTH and MOH. The Eurolight project, supported by the EC European Agency for Health and Consumers of the European Commission, was a data-gathering exercise undertaken primarily to inform health policy on headache disorders in the European Union (EU). This manuscript reports the principal findings with regard to the personal impact of headache, which translates into public ill-health. Our findings on financial cost and societal impact are published elsewhere [19].

## Methods

The study was of cross-sectional design and used modified cluster sampling. There was pragmatic regard to convenience in the selection of countries, and areas within those countries, from which samples were drawn. Using the same questionnaire, translated into the local languages, surveys were conducted in 10 countries: Austria, France, Germany, Ireland, Italy, Lithuania, Luxembourg, Netherlands, Spain and United Kingdom (UK). The full methodology is described elsewhere [20]; below we present brief details.

### Project organization

The Eurolight project was a collaboration of 25 groups from 15 different countries: two public bodies (CRP Santé, Luxembourg, and Regione Lombardia—Sanità, Italy); clinicians from 11 hospitals; the professional European Headache Federation (EHF); nine European patients’ organizations including the European Headache Alliance (EHA); the World Headache Alliance (WHA); and *Lifting The Burden* (LTB), a UK-registered nongovernmental organization directing the Global Campaign against Headache in official relations with WHO.

### Ethics

The National Ethics Committee of Luxembourg gave overall approval of the protocol. Further approvals were obtained from national or local ethics committees wherever needed as the methods for recruitment of participants differed between countries. Similarly, data protection approvals were obtained centrally in Luxembourg and at country levels in compliance with national and European privacy laws.

In each country, prospective participants received a written information sheet explaining the project and enquiry, and their purpose.

### Questionnaire

The development, content and validation of the structured questionnaire have been previously described [21]. The original English version was translated into Dutch, French, German, Italian, Lithuanian, Luxembourgish, Portuguese and Spanish according to LTB’s standardized translation protocol for lay documents [22].

Demographic questions were followed by neutral screening questions for headache (“Have you ever had a headache?” and “Have you had a headache during the last year?”) and, in those screening positively, by headache-diagnostic questions and several question sets addressing impact.

### Diagnosis

The diagnostic questions were imported, with linguistic adaptations as necessary, from the burden-of-headache questionnaire developed by LTB for population-based surveys (the HARDSHIP questionnaire [23]). When participants reported more than one headache type, questions were directed to the one identified as the most bothersome. Diagnoses were made by computerized algorithm also developed by LTB specifically for this questionnaire [23]. The algorithm first identified, and separated, participants reporting headache on ≥15 days/month (of whom additional questions enquired into medication use), then to the remainder applied ICHD-II criteria [24] for migraine, TTH, probable migraine and probable TTH in that order. Thus a diagnosis of TTH trumped probable migraine [24]. In the analyses, migraine and probable migraine were considered together, as were TTH and probable TTH [25,26]. Probable MOH was assumed to be the diagnosis when headache frequency was ≥15 d/mo, duration was >4 h, the question “Do you usually take medication to treat your headaches” was answered “yes”, and frequency of acute medication use was ≥15 d/mo if the medication was simple analgesics only and ≥10 d/mo if any other (compound analgesics, opioids, triptans and/or ergots). A diagnosis of probable MOH trumped all other diagnoses.

The questionnaire did not attempt to diagnose relatively rare headache disorders such as the trigeminal autonomic cephalalgias; even in very large samples, no more than a very few cases would occur.

### Sampling and data collection

We adopted sampling methods which varied between countries according to what was feasible. Again, these are fully described elsewhere [20]. The sample drawn from Lithuania was population-based, but those in other countries were to varying degrees less so (Table 1). Additional surveys in Spain and the Netherlands, and the only survey in Ireland, were performed among members of the national headache patients’ organizations. The samples generated from these were inevitably biased, and the data from them are not reported here.

**Table 1:** Summary of data collection methods in each country, excluding samples drawn through patients’ organizations

Country*	**Sample size (n)**	**Methods**
Lithuania	1,137	Sample drawn from inhabitants of Kaunas city and Kaunas region using Residents’ Register Service, reflecting age (in range 18–65 y) and gender composition of Lithuania and proportions living in rural (33%) or urban (67%) areas. Data collection face-to-face, conducted by medical students “cold-calling” door-to-door.
Luxembourg	6,498	Sample aged 18–65 y, stratified for age, gender, region and nationality, drawn from general population via national social security registry (IGSS). Questionnaires distributed and returned by post. Reminders sent one month later to non-responders.
Spain	1,700	Random sample of employees of various companies operating in national postal services in 10 areas of Spain, stratified to be representative of general working population with regard to gender, age (within range 18–65 y) and education. Ten occupational health physicians delivered and took return of questionnaires. One reminder by telephone to non-responders.
Germany	3,000	Random urban (50%) and rural (50%) samples aged 18–65 y from general population listings supplied by local municipal authority. Questionnaires distributed and returned by post. No reminders sent.
Italy	3,500	Random urban (70%) and rural (30%) samples drawn from general population using listings supplied by Azienda Sanitaria Locale of Pavia, stratified with regard to gender, age (in range 18–65 y) and education. Questionnaires distributed and returned by post. No reminders sent.
France	2,400	Consecutive patients aged 18–65 y attending any of cooperative of 80 general practitioners (GPs) on a pre-specified day. Questionnaires to be completed and returned immediately or later by post. One reminder by email after one week to non-responders.
Austria	up to 6,000	Up to 10 consecutive patients aged 18–65 y visiting any of 400 GPs and 200 neurologists for any reason on a pre-specified day. Questionnaires to be completed and returned later. One reminder after one month to non-responders.
Netherlands	unknown	Survey conducted by TNS-NIPO, a market research company with access to a population sample of 200,000, representative with regard to gender, age (in range 18–65 y), region and education. Questionnaire distributed by internet, to be completed on-line. Study stopped when >2,000 received back.
UK	720	Modified population-based sampling attempted through 12 GP practices in 11 areas (in UK, virtually all residents are registered with local GP). Questionnaire given to consecutive patients aged 18–65 y attending for any reason over a period of time, to be completed and returned immediately, or later by post.

*Listed in descending order corresponding to how well the sample was population-based.

### Non-responder study

Participation rates were low in some countries and we recognized that questionnaires were more likely to be completed and returned by those most affected by headache (a form of participation bias referred to as interest bias [26]. Therefore, studies of non-responders were performed in Luxembourg, Italy, Netherlands and Germany. In Italy, non-responders were invited by means of advertisements in local newspapers to complete a short questionnaire on the website of Centro Italiano di Ricerche Neurologiche Applicate (CIRNA) (http://www.cefalea.it). In the other countries, non-responders were selected randomly and called by telephone. All were asked whether headache had occurred ever and, if so, during the preceding year and how often.

### Data management and analysis

All completed questionnaires were transferred electronically to the data-management centre at CRP-Santé. Double-data-entry and reconciliation of inconsistencies were employed as quality-control procedures.

For reasons given above, analyses included nine countries only, ignoring those samples derived from the memberships of national patients’ organizations.

Statistical analyses were performed at CRP-Santé using SAS version 9.2, and the further calculations of results described in the text were performed by LJS, using Excel version 14.0.6123.5001. Analyses presented here are essentially descriptive. The study was neither designed nor powered to generate prevalence estimates for individual countries but, through modified cluster sampling, to produce estimates for the European Union. Because they were not taken to be nationally representative, country-derived data were pooled without weighting according to country population size.

## Results

From these nine countries, 8,271 correctly completed questionnaires were analysed. Participation-rates were not calculable in Austria and the Netherlands, where the denominators were unknown, but elsewhere varied from 10.6% to 58.8% (Table 2). Participants had a moderate female bias (58%) and mean age of 43.4 y; almost two thirds (65%) were employed and nearly three quarters (72%) were married or living with a household partner (Table 2).

**Table 2:** Numbers of participants, participation rates and demographic characteristics of samples per country

**Country**	**Participants (n)**	**Denominator (N)**	**Participation rate (n/N) (%)**	**Gender (% female)**	**Age (y) (mean [SD])**	**Employed or self-employed (%)**	**Married or living with partner (%)**
Austria^†^	644	unknown, but not >6,000	not calculable	70	48.8 [16.0]	57	75
France^†^	876	2,400	36.5	68	50.2 [16.7]	52	80
Germany	318	3,000	10.6	57	44.6 [12.5]	70	65
Italy	487	3,500	13.9	58	43.4 [12.6]	68	92
Lithuania	573	1,137	50.4	59	40.9 [13.8]	65	67
Luxembourg	1,833	6,498	28.2	59	40.5 [12.7]	67	71
Netherlands	2,414	unknown	not calculable	50	42.6 [13.2]	69	69
Spain	999	1,700	58.8	59	42.7 [11.9]	83	69
UK^†^	127	720	17.6	65	48.0 [18.3]	54	67
Overall	8,271		27.5*	58	43.4	65	72

^**†**^Sample derived from health-care setting (see Table 1). *Excluding Austria and Netherlands.

In the non-responder studies there were 1,007 participants (Germany 260, Italy 202; Luxembourg 357; Netherlands 188; 51% female overall). Participation rates in these studies were generally high (Germany 80%; Luxembourg 87%; Netherlands 72%), although in Italy the denominator was unknown.

The samples from six countries (n = 6,624) were derived from the general population, those from Austria, France and UK (n = 1,647) were derived from health-care (but not headache-specific) settings (see Table 1). Lifetime prevalence of any headache (unadjusted for gender or age) was 91.3% in the general population samples, 91.2% in the health-care samples (91.3% overall; n = 8,271). Lifetime prevalence by gender (overall) was 87.5% in males and 94.3% in females; in males, life-time prevalence peaked at 92.8% in the age range 20–30 y, whereas in females there was a plateau at 96-97% in the age range 20–50 y. The 1-year prevalence was 79.6% overall, 71.1% in males (with a peak of 81.3% in the age-range 30–40 y) and 86.0% in females (peaking at 92.7% in the age-range 20–30 y). In both genders, 1-year prevalence declined substantially after age 60 y (48.2% in males, 61.9% in females).

By comparison, in the non-responder studies the life-time prevalence of headache was slightly lower at 86% (95% CI: 83.9-88.1%), but the 1-year prevalence was considerably lower at 64% (95% CI: 61.0-67.0).

Table 3 shows the unadjusted 1-year prevalences of specific headache types based on the reported most bothersome headache. In males, migraine prevalence peaked at 33.5% in the age range 30–40 y; in females there was a plateau at 37-40% in the age range 20–60 y. After age 60 y, prevalence fell dramatically in both genders (males: 12.2%; females 22.3%). TTH showed limited variation in both genders between 20 and 60 y, but peaked between 20 and 30 y (males: 46.6%; females: 41.2%).

**Table 3:** One-year prevalences (unadjusted) of specific headache types in the main sample (n = 8,271)

**Diagnosis**	**Prevalence (% [95% CI])**		
	**Overall**	**Male**	**Female**
Any headache	79.6 [78.7-80.5]	71.1 [69.6-72.6]	86.0 [85.0-87.0]
All migraine	36.6 [35.6-37.6]	26.9 [25.4-28.4]	43.6 [42.2-45.0]
Definite migraine	22.2 [21.3-23.1]	14.8 [13.6-16.0]	27.7 [26.4-29.0]
Probable migraine	14.3 [13.6-15.1]	12.1 [11.0-13.2]	15.9 [14.9-16.9]
All TTH	37.6 [36.6-38.6]	40.7 [39.1-42.3]	35.7 [34.3-37.1]
Definite TTH	30.8 [29.8-31.8]	33.8 [32.2-35.4]	28.9 [27.6-30.2]
Probable TTH	6.8 [6.3-7.3]	6.9 [6.1-7.7]	6.8 [6.1-7.5]
Headache on ≥15 d/mo	7.6 [7.0-8.2]	4.9 [4.2-5.6]	9.5 [8.7-10.3]
Probable MOH	3.3 [2.9-3.7]	1.8 [1.4-2.2]	4.3 [3.7-4.9]

*CI*: confidence interval; *TTH*: tension-type headache; *MOH*: medication-overuse headache.

Adjustment for age and gender was problematic because of uncertainties about the demographics of any reference population. In the European Union, the ratio of males to females among adults aged 18–65 y is very close to 1.00 [27] on which basis the gender-adjusted 1-year prevalences were: any headache 78.6%; all migraine 35.3%; all TTH 38.2%, all headache on ≥15 d/mo 7.2%; probable MOH 3.1%.

Further analyses of prevalence are not presented because the emphasis of Eurolight was on impact. Regarding symptom burden, irrespective of diagnosis, mean headache frequency was 4.7 d/mo in males and 6.3 d/mo in females; 16.6% of males and 8.7% of females with headache had infrequent episodes (<1 d/mo), while 50.0% of males and 42.8% of females with headache experienced episodes on 1–3 d/mo. Thus, in the remaining one third (33.4%) of males and one half (48.5%) of females (41.7% overall), headache was a weekly or more frequent occurrence. In the non-responder studies by comparison, only 24.7% (158 of 639) with headache in the last year reported this level of frequency.

**Figure 1:**
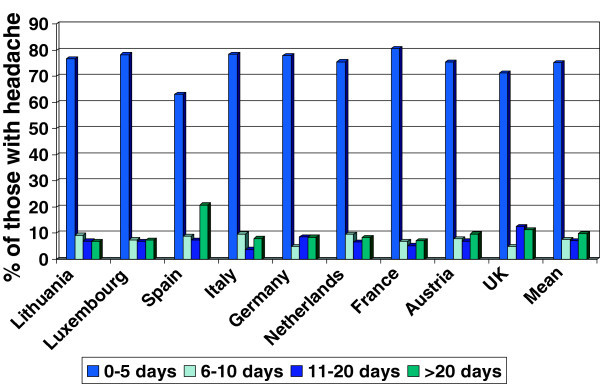
Personal impact of headache assessed by HALT index (days lost in preceding 3 months), by country.

Personal impact was assessed in part by a set of seven questions, responses to which are shown in Table 4. In most of these questions, migraine was 2–3 times as likely as TTH to be associated with an adverse response, and probable MOH much more so (*eg*, 32.9% for question 2; 33.7% to question 3; 49.6% to question 5; 30.0% to question 7). The clear exception was question 6, answered similarly (adversely by about 10%) by those with migraine and TTH (and by 21.5% with probable MOH). Personal impact in terms of lost useful time was measured by the HALT index [28]. In five questions this captured, over the preceding 3 mo and attributable to headache: a) workdays lost completely, or with productivity reduced to <50% of expected; b) the same for days of household work or chores, c) days on which family, social or leisure activities were lost. Summing the responses to all five questions provided overall estimates of individual impact. These data were surprisingly uniform across countries (Figure 1). Analysis on this basis by diagnosis and gender (Table 5) found that impact was always higher in females regardless of diagnosis. Impact was higher for those with migraine than for those with TTH, with substantial proportions of the former (17.7% of males and 28% of females) losing >10 d in 3 mo (*ie*, >10% of available days). For those with probable MOH, impact was much higher, to the extent that half (44.7% of males, 53.7% of females) reported the loss of >20 d.

**Table 4:** Personal impact of headache assessed by seven questions

**Question**	Proportion responding adversely* (%)		
	**Overall**	**Male**	**Female**
1. Have your headaches interfered with your education?	9.2	7.9	9.9
2. Do you believe your headaches have made you less successful in your career?	7.7	7.0	8.1
3. Have your headaches resulted in reduced earnings?	8.4	8.0	8.7
4. Do you avoid telling people that you have headaches?	31.4	30.1	32.1
5. Do you feel that your employer and work colleagues understand and accept your headaches?	36.3^†^	38.9^†^	34.9^†^
6. Do you feel that your family and friends understand and accept your headaches?	10.8	10.5	10.9
7. Taking into account everything you do to treat your headaches, do you feel you are in control of your headaches?	13.5	13.0	13.7

*“Yes” to questions 1–4; “no” to questions 5 and 6; “rarely” or “never” to question 7.

^**†**^Of those to whom the question was applicable (*ie*, having headache and being employed).

**Table 5:** Personal impact of headache assessed by HALT index (days lost in preceding 3 months), by diagnosis and gender

**Diagnosis and gender**	**Days lost (% [95% CI])**			
	**0-5 days**	**6-10 days**	**11-20 days**	**>20 days**
Migraine				
Males	73.0 [±3.0]	9.3 [±2.0]	7.8 [±1.8]	9.9 [±2.0]
Females	57.7 [±2.3]	14.4 [±1.6]	12.2 [±1.5]	15.8 [±1.7]
Tension-type headache				
Males	92.0 [±1.5]	3.3 [±1.0]	2.2 [±0.8]	2.5 [±0.8]
Females	87.5 [±1.7]	5.4 [±1.1]	3.4 [±0.9]	3.7 [±1.0]
Probable medication-overuse headache				
Males	34.0 [±13.5]	10.6 [±8.8]	10.6 [±8.8]	44.7 [±14.2]
Females	31.4 [±8.3]	6.6 [±4.4]	8.3 [±4.9]	53.7 [±8.9]

*CI*: confidence interval.

Analysis of HALT by categorization in this way does not yield intuitive information about the amount of time actually lost; neither does it distinguish between lost workdays, lost housework days and lost social days. For these purposes, days lost were analysed as continuous data (Table 6).

**Table 6:** Personal impact of headache assessed as headache-attributed lost work, housework and social days in preceding 3 months, by diagnosis and gender

**Diagnosis and gender**	**Days lost in preceding 3 months**											
	**Workdays**				**Housework days**				**Social days**			
	**Mean [SD]**	**Range**	**Median**	**Upper quartile**	**Mean [SD]**	**Range**	**Median**	**Upper quartile**	**Mean [SD]**	**Range**	**Median**	**Upper quartile**
Migraine	3.2 [8.6]	0-120	0	3	4.6 [9.2]	0-115	1	6	2.1 [5.0]	0-90	0	2
Males	2.9 [9.4]	0-120	0	2	3.3 [8.7]	0-100	0	2	1.7 [4.8]	0-90	0	2
Females	3.4 [8.3]	0-90	0	3	5.2 [9.4]	0-115	2	6	2.3 [5.1]	0-90	0	3
Tension-type headache	1.0 [5.7]	0-90	0	0	1.3 [5.7]	0-90	0	0	0.6 [3.9]	0-90	0	0
Males	1.0 [6.0]	0-90	0	0	1.0 [5.5]	0-90	0	0	0.6 [3.9]	0-90	0	0
Females	1.0 [5.5]	0-90	0	0	1.6 [5.8]	0-90	0	1	0.7 [3.8]	0-86	0	0
Probable MOH	14.2 [26.1]	0-180	3	17	21.4 [26.7]	0-155	12	30	9.0 [16.2]	0-90	3	10
Males	15.1 [32.7]	0-180	2	10	20.0 [31.5]	0-150	6	24	9.7 [18.6]	0-90	2	10
Females	13.9 [23.3]	0-155	5	20	21.9 [25.3]	0-155	14	30	8.8 [15.3]	0-90	3	10

*MOH*: medication-overuse headache; *SD*: standard deviation.

For migraine, mean lost workdays were about 1 day/month and mean lost housework days were the same in males but approached 2 days/month in females. For TTH, lost days were generally about 1 day per 3 months, with lost housework days somewhat higher (1.6) in females. However, the distributions in these disorders were not normal, with medians of 0 for both disorders in both genders (and upper quartiles of 0 for TTH) despite ranges of 0–90 or higher. For these disorders, a small minority of participants accounted for a highly disproportionate part of the reported burden. We identified 158 participants (2.4% of those with headache) who gave responses of ≥45 days to at least one of lost work, housework or social days, of whom over one third (59) had probable MOH. Of the others, 74 had migraine (2.4% of those with this diagnosis) and 24 had TTH (0.7% of those with TTH). One had unclassified headache. Table 6 shows much higher levels of lost workdays in probable MOH according to the means, but still with an overall median of only 1 day/month. Lost housework days were higher still, about 23% of all days according to the means, but with medians reflecting about half of this.

There was a direct enquiry into interictal burden. First, participants were instructed to think carefully about the last day when they did not have headache. Then they were asked three questions specifically about that day:

• “On that day, were you anxious or worried about your next headache episode?”

• “On that day, was there anything you could not do or did not do because you wanted to avoid getting a headache?”

• “On that day, did you feel completely free from all headache-related symptoms?”.

All were answerable “yes” or “no”, and the analysis in Table 7 sums the adverse responses (“yes” to the first two questions, “no” to the third).

**Table 7:** Interictal burden assessed by three questions

**Diagnosis and gender**	**Number of adverse responses given (%)**			
	**0**	**1**	**2**	**3**
Migraine				
Males	17.3	70.8	9.5	2.4
Females	15.1	71.5	11.4	2.0
Tension-type headache				
Males	18.7	76.3	4.7	0.4
Females	14.9	81.2	3.6	0.4
Probable medication-overuse headache				
Males	15.0	60.0	20.0	5.0
Females	18.2	50.5	24.4	6.8

Gender differences were small in this analysis, while the differences between migraine and TTH suggested somewhat – but not markedly – greater interictal burden in the former. Analyses by headache frequency (not shown) revealed surprisingly little effect; for example, among participants with migraine headache on 10–14 d/mo, 15.5% recorded no adverse responses, 61.6% gave one, 18.6% gave two and only 4.3% answered adversely to all questions. These findings were very similar to those for probable MOH, occurring by definition on ≥15 d/mo.

Seven questions enquired into impact on others, in three areas of family life (Table 8). In these there were some gender effects. For question 1, females with migraine answered “yes” four times as often as males, and females with probable MOH three times as often as males. Females with migraine answered “yes” to question 5 about twice as often as males. In this and all other questions except 4, males with probable MOH answered “yes” rather more often (up to 1.5 times) than females. In most cases, likelihood of responding “yes” was strongly correlated with reported headache frequency, although numbers were small for some of these analyses.

**Table 8:** Impact of headache on others assessed by seven questions in three areas of family life

**Question**	**Proportion responding “yes” (%)**		
	**Migraine**	**TTH**	**Probable MOH**
**Relationships, love life and family planning**			
1. Have your headaches affected your family planning (fewer children, or avoided having children)?	5.5^†^	1.1^†^	21.1^†^
2. During the last 3 months, have your headaches caused difficulties in your love life?	17.8^†^	5.5^†^	48.6^†^
3. Have your headaches caused a relationship to break down (separation or divorce)?	0.7	0.3	7.0
**Children**			
4. During the last 3 months, have your headaches caused one or more of your children to miss school?	1.7^†^	1.5^†^	5.0^†^
5. During the last 3 months, have your headaches prevented you from caring for your children?	18.2^†^	7.9^†^	50.0^†^
**Household partner**			
6. During the last 3 months, have your headaches caused your partner to lose time from work?	2.6^†^	0.9^†^	10.7^†^
7. During the last 3 months, have your headaches caused your partner to miss social activities?	9.1^†^	2.9^†^	24.6^†^

*TTH*: tension-type headache, *MOH*: medication-overuse headache.

^**†**^Of those to whom the question was applicable (*ie*, having headache and a household partner and/or children).

## Discussion

This was a very large and organizationally complex study, involving multiple collaborating partners (academic and lay) in ten countries. We made pragmatic methodological compromises in order to complete it.

A considerable strength of the study was the use everywhere of the same instrument (questionnaire). Not only that, but the questionnaire was derived from the HARDSHIP questionnaire, already used by LTB in many different countries, cultures and translations [23]. The very neutral screening questions are expected to have led to better ascertainment, and therefore higher headache prevalence estimates, than questions requiring a certain degree or frequency or severity [26]. Diagnoses were made according to a standard algorithm also developed and widely used by LTB [23]. Nevertheless, the nature of this study did not allow diagnostic validation in each of the translations used here, so diagnostic accuracy was not directly assessed. While it was also a strength of the study that it enquired simultaneously into migraine, TTH and MOH, so that these three disorders could be compared with regard to prevalence and impact, this diagnostic uncertainty must be taken into account in doing this.

The different sampling methods employed by the countries produced samples that varied in their representativeness of the general population. This was both a strength, in that it enables comparisons between differently sourced samples (although that was not a purpose of the analyses presented here), and a weakness in that representativeness is an absolute requirement for external validity. In fact, there were no obvious differences between the population- and health-care samples, while it was readily apparent that headache was more prevalent, and had higher impact, in the samples generated from membership lists of lay groups in Spain, Ireland and the Netherlands. We expected this, and here excluded consideration of the lay samples. Among the population and health-care samples, drawn from nine countries only, the UK sample showed clear evidence of biases, with a high proportion having frequent headaches, but it was a very small contributor (n = 127) to the very large total of 8,271 participants. Despite this large number, the participation rate was low: 27.5% overall (excluding Austria and the Netherlands, where it was unknown), and only 10.6% in Germany and <20% in two other countries. This means unrecognised biases were likely. Low participation rates might not be a problem if the reasons for not responding were unrelated to headache or its impact, but this was probably not so. The most likely bias was that people affected by headache, and, particularly, people who perceived themselves to be badly affected, had more interest in responding and were therefore over-represented. This so-called interest-bias was detectable in the gender distribution: nearly 60% of the sample were women, in whom headache is rather more common.

The non-responder study was conducted to provide insight into this bias, and it did so. Whereas lifetime headache prevalence was 91% in the main study (in both population and health-care samples), in the non-responder study, with a high participation rate, it was not dissimilar at 86%. One-year prevalence on the other hand did indicate interest bias: 79.6% (unadjusted) in the main study *versus* 64% in non-responders. If a correction were to be made, we could re-estimate the 1-year prevalence at 68.3% by taking the weighted average of 79.6% (representative of the 27.5% of the source population who responded initially (Table 2)) and 64% (representative of the 72.5% of the source population who did not). The difference of 11.3% between this re-estimate and the original suggests that interest bias may have led to overestimation by some 14% (relative, calculated as 11.3/79.6*100), which is a moderate influence. Frequency comparisons between participants in the main study and initial non-responders provided more evidence of interest-bias: 41.7% of the former with headache in the last year, but only 24.7% of the latter, reported headache as a weekly or more frequent occurrence.

While the large sample size meant that measures of statistical uncertainty (*eg*, confidence intervals) were small, clinical uncertainties were therefore far from negligible. The estimated 1-year prevalence of migraine (gender-adjusted: 35.3%) is outside the range of other published studies [2], even recent LTB studies which, with very careful case-ascertainment, have generally found migraine to be more prevalent than previously reported (*eg*, 20.8% in Russia [10]; 25.6% in India [unpublished]). Allowance for an interest-bias-related overestimation margin of 14% (see above) would reduce the estimate to 30.4%, which is still high. In fact this may not be attributable to interest-bias – at least not entirely – since the country with the highest participation rate (Spain: 58.8%), and therefore least vulnerable to interest-bias, produced an even higher estimate of 35.4%. A factor is that migraine and probable migraine were combined, which has been argued to be correct in epidemiological studies provided that a diagnosis of probable migraine is trumped (as here) by a diagnosis of TTH [25,26]. On the other hand, the estimated 1-year prevalence of TTH (gender-adjusted: 38.2%) is close to the reported global mean of 42% [2], which does not suggest any large effect (if any) of questionnaire-misdiagnosis of TTH as migraine, especially since the focus on the most bothersome headache in those with two or more distinct headache types (again a pragmatic solution [26]) did mean that TTH would not always be recognized. It might be supposed that the prevalence of TTH in those with migraine, but not reporting the former because it was less bothersome, was the same as in those without, which would inflate the TTH prevalence estimate by 13.5% to 43.4%, very close to the reported global mean.

The high prevalence estimates for all headache on ≥15 d/mo (7.2%) and for probable MOH (3.1%) were also, very probably, influenced by interest-bias. They are not, however, outside the range of national studies (in Russia, 10.4% and 7.1% respectively were reported [10,26]). These disorders cause high disability at individual level (discussed further below); even after discounting to allow for likely overestimation, these prevalence estimates are indicative of very substantial population ill-health. They should give rise to considerable political concern, and remedial action.

Estimates of individual impact, to the extent that they are dependent on prevalence estimates, may therefore not be quantitatively exact, but this does not mean they are not indicative. The general impact gradient (probable MOH > migraine > TTH) is reassuringly as expected, as are the gender-related differences. Symptom burden is difficult to quantify objectively. Duration and intensity of headache are dimensions of symptom burden, but apt to be misleading (subject to the effects of any treatments taken and, in reporting, to recall bias). They are not reported here, but will be included in another manuscript focusing on headache on the day prior to the enquiry (“headache yesterday”), which minimises the effects of erroneous recall. As to frequency, while >40% of those with headache reported it as at least a weekly occurrence (with the non-responder study suggesting this was an overestimate), this alone, although of interest, is not a good measure of burden – as is shown by the loose relationship between frequency and interictal burden (Table 7). As a consequence of symptom burden, the penalty in lost useful time is more readily measurable (Tables 5 and 6). Nearly one fifth of males and over a quarter of females with migraine reported the loss of >10% of available days, as did over half of males and nearly two thirds of females with probable MOH. The inescapable financial implications of such losses, reported in detail elsewhere [19], again show a clear gradient at personal level of probable MOH > migraine > TTH.

Something more should be said about lost time estimation. It is a well-validated measure of burden [29], but with a tendency to break down at the very high end. Our recorded ranges of 0–90 and higher clearly signalled an element of double counting: neither the workdays nor the housework days lost in three months can exceed 90 (in fact, few people can claim >65 workdays in 3 months). However, only 2.4% of participants with headache gave responses of ≥45 days to any of the questions enquiring into lost work, housework or social days, and over one third of these had probable MOH, so it was not a significant problem in the sense of being influential. And it should be assumed that these people, while not being numerically accurate, were indeed expressing what they felt was heavy burden. Again, enquiry into headache yesterday can largely obviate this problem.

The aspects of personal impact identified in Table 4 are worth dwelling on, because they signal effects that are constant and/or cumulative, not merely present during headache episodes. Such consequences are serious impositions on life, particularly the effects on education, career and earnings reported by 8-9%. Similar comments can be made of those aspects of personal impact reported in Table 8, which in addition affect others than the people who actually experience headache. Especially notable are the 18% of parents with migraine and 50% with probable MOH whose children have not, on at least one occasion in the preceding 3 months, received the care they might expect. One in 40 people with migraine and one in ten with probable MOH have caused their partners to lose work-time in the last 3 months. These and many other aspects of interictal burden will be reported in more detail in a future publication. For many of them, there is little or no evidence from previous studies for comparison. The analysis to be done needs to explain, if it can, why interictal burden assessed by direct enquiry correlated poorly with headache frequency.

## Conclusions

In conclusion, Eurolight should not be seen as a primary source of prevalence estimates, for which it was neither intended nor designed. The key findings, while subject to some diagnostic uncertainty regarding headache type and to moderate interest-bias, reveal that the common headache disorders have very high personal impact. The multiple components of this extend both beyond active headache episodes and beyond the people who actually experience headache. The level of this impact, and its pervasiveness, taken together with estimates of huge financial cost [19], have important implications for health policy in Europe, since they are indicators not only of much public ill-health and unmet health-care need but also of high but reducible socioeconomic burden.

## Competing interest

TJS, LJS and ZK are directors and trustees of *Lifting The Burden*, a charitable organization whose purpose, pursued in official relations with the World Health Organization, is to reduce the burden of headache worldwide. There were no conflicts of interest relating to the content of this manuscript.

## Authors’ contributions

CA was project leader. All authors contributed to project design and development of the methodology, which were led by LJS and TJS. CA, ZK, JML, CL, ML-M, DR, ERdlT and CT contributed to data acquisition. Analysis and data interpretation were performed by JB, LJS and TJS. The article was drafted by TJS with input from LJS, and revised for intellectual content by CA, ZK, JML, CL, ML-M, DR and CT. All authors reviewed and approved the final manuscript.
